# Metabolic remodeling and cardiac functional adaptation mediated by dapagliflozin in patients with acute heart failure: a retrospective cohort study

**DOI:** 10.3389/fphar.2026.1913021

**Published:** 2026-08-10

**Authors:** Feng Liu, Xingjun Liu, Yuping He, Guobin Liu, Ting Lan, Zeng Zhao, Faming Yang

**Affiliations:** 1 Department of Cardiology, Jintang County First People’s Hospital, Chengdu, China; 2 Department of Cardiology, Shifang City People’s Hospital, Deyang, China

**Keywords:** acute heart failure, ardiac function, dapagliflozin, metabolic remodeling, SGLT2 inhibitors, ventricular remodeling

## Abstract

**Aims:**

Grounded in the “myocardial metabolic starvation” hypothesis, this study evaluated the pharmacological effects of dapagliflozin on serum metabolic profiles, ventricular remodeling, and cardiac function in patients hospitalized with acute heart failure (AHF), and explored the associations between circulating metabolic alterations, ventricular remodeling, and cardiac functional parameters.

**Methods:**

This single-center retrospective cohort study included 50 AHF patients (25 pairs) after 1:1 propensity score matching. The dapagliflozin group received standard care plus dapagliflozin 10 mg once daily for 6 months, while the control group received standard care alone. Changes in circulating metabolic parameters, including fasting blood glucose, lactic acid, free fatty acids, and ketone-related metabolites (β-hydroxybutyrate and acetoacetic acid), as well as glycolytic enzyme activity (pyruvate kinase), were compared between groups. Echocardiographic parameters, 6-min walk test (6 MWT) distance, B-type natriuretic peptide (BNP), and major adverse cardiovascular events (MACE) were also assessed.

**Results:**

The dapagliflozin group showed greater reductions in circulating metabolic parameters, particularly fasting plasma glucose, free fatty acids, β-hydroxybutyrate, and acetoacetic acid, compared with controls (all *P* < 0.05). Left ventricular end-systolic diameter (37.29 ± 4.07 vs. 43.16 ± 4.41 mm) and end-diastolic diameter (45.17 ± 5.02 vs. 52.63 ± 6.20 mm) improved more markedly in the dapagliflozin group (both *P* < 0.001). left ventricular ejection fraction (48.36% ± 4.88% vs. 43.85% ± 4.90%, *P* = 0.002), BNP (116.45 ± 12.15 vs. 214.50 ± 20.36 pg mL^-1^, P < 0.001), and 6 MWT distance (385.56 ± 51.42 vs. 314.78 ± 42.17 m, P < 0.001) were also significantly superior in the dapagliflozin group. FFA reduction correlated positively with LVEDD reduction (r = 0.638, P < 0.001). Multiple regression analysis identified ΔFFA (β = −0.412, *P* = 0.008) and Δβ-HB (β = −0.385, *P* = 0.012) as independent predictors of ΔLVEF improvement. MACE incidence was numerically lower in the dapagliflozin group (8.0% vs. 32.0%, *P* = 0.076).

**Conclusion:**

In this small retrospective cohort, early in-hospital initiation of dapagliflozin was associated with favorable changes in circulating metabolic profiles, ventricular remodeling, and cardiac function in patients with acute heart failure. These findings suggest potential therapeutic benefits for ventricular remodeling, but require confirmation in larger prospective studies.

## 1 Introduction

Heart failure (HF) is a complex clinical syndrome defined by impaired ventricular systolic and/or diastolic function, resulting in a mismatch between cardiac output and systemic metabolic demands. Its etiological spectrum encompasses primary cardiovascular diseases—including myocardial infarction, chronic ischemia, dilated/hypertrophic cardiomyopathy, and valvular lesions—as well as systemic contributors such as diabetes mellitus, hypertension, hyperthyroidism, and viral myocarditis. Among acute precipitating factors, respiratory tract infections remain the most common trigger for decompensation. Symptomatically, patients suffer from progressive dyspnea, fatigue, muscle weakness, and exercise intolerance; when cerebral and peripheral perfusion become insufficient, dizziness and functional decline ensue, collectively driving high hospitalization rates and poor long-term outcomes (Pfeffer et al., 2019; Skrzypek et al., 2018).

For decades, the pharmacological backbone of HF treatment has relied upon neurohormonal antagonism—namely β-blockers, ACEIs, and ARBs. While these agents indisputably slow disease progression, they leave a substantial residual risk of cardiovascular death and readmission, underscoring a clear therapeutic ceiling that novel pharmacological strategies must overcome (Castiglione et al., 2022; Savarese et al., 2022). This persistent gap has redirected research efforts toward identifying pathophysiologically distinct, drug-targetable pathways beyond the classical renin–angiotensin–adrenergic axis (Borlaug, 2020; González et al., 2018).

The advent of sodium-glucose cotransporter 2 (SGLT-2) inhibitors has heralded a paradigm shift in cardiovascular pharmacotherapy. Originally designed as glucose-lowering agents, this class has consistently demonstrated unexpected, robust cardiovascular benefits across landmark outcome trials (Crespo-Leiro et al., 2018; McMurray et al., 2019a; Nassif et al., 2021). Dapagliflozin was the first SGLT-2 inhibitor to receive regulatory approval for HF with reduced ejection fraction (HFrEF), irrespective of diabetic status, based on the DAPA-HF trial. Its indication has since expanded to cover the full LVEF spectrum, including HF with preserved ejection fraction (HFpEF), across major global regions. Beyond symptomatic relief, dapagliflozin exerts pleiotropic pharmacological actions—including blood pressure reduction, lipid profile modulation, and direct myocardial protection—that translate into significantly lowered cardiovascular mortality and HF-related rehospitalizations (McMurray et al., 2019a; Nassif et al., 2021).

Despite these well-documented macro-level benefits, the precise *in-vivo* pharmacological mechanisms driving these effects remain incompletely understood, as large RCTs were not designed to capture serial metabolic changes at the tissue or circulating level. Converging preclinical evidence points to a “myocardial metabolic starvation” model, wherein the failing heart loses substrate flexibility and becomes energy-depleted (Sharif et al., 2025; Petrie et al., 2020). However, translational pharmacological data—specifically, the concurrent quantification of circulating fatty acids, ketone bodies, and cardiac reverse remodeling in the acute HF setting—are notably scarce. To address this gap, we designed this retrospective cohort study to test the hypothesis that adding dapagliflozin to standard therapy in patients hospitalized with AHF induces alterations in circulating substrate profiles involving glucose, lipid, and ketone metabolism, and that these circulating metabolic changes are associated with ventricular remodeling and cardiac functional parameters. By examining this metabolic–structural–functional pharmacological axis, we seek to provide mechanism-oriented, real-world clinical evidence for dapagliflozin’s cardioprotective actions in a Chinese patient population.

## 2 Methods

### 2.1 Study design and patients

This study was designed as a single-center, retrospective, baseline-matched cohort study to evaluate the efficacy and safety of dapagliflozin in patients with acute heart failure (AHF). Following the U.S. Food and Drug Administration (FDA) approval of dapagliflozin for heart failure in 2020, our hospital integrated this medication into the routine treatment protocol for AHF at the end of 2021.

A total of 73 patients diagnosed with and treated for AHF at our institution between June 2021 and June 2022 were retrospectively screened for eligibility. Patients admitted prior to the implementation of the new protocol served as the control group (receiving conventional anti-heart failure therapy), whereas patients admitted after its implementation were designated as the dapagliflozin group (receiving conventional therapy supplemented with dapagliflozin). After propensity score matching (PSM) based on baseline demographic and clinical characteristics, 50 patients were finally analyzed, with 25 patients in each group. The dapagliflozin group consisted of 25 patients (14 males and 11 females; mean age: 53.50 ± 6.71 years), and the control group consisted of 25 patients (13 males and 12 females; mean age: 55.12 ± 7.33 years). Baseline demographic and clinical characteristics were highly comparable between the two groups (all P > 0.05). Propensity score matching was performed retrospectively after data extraction, using baseline characteristics collected prior to treatment initiation, to ensure that the matching process did not influence outcome assessment. To ensure the validity of the propensity score model, only baseline covariates available before treatment initiation were included in the logistic regression model used to calculate propensity scores. No post-treatment variables, follow-up measurements, or clinical outcomes were included in the matching procedure.

Clinical data were extracted from electronic medical records by two independent investigators (X.J.L. and G.B.L.) using a standardized case report form. Both investigators were blinded to the group assignment at the time of data extraction. Discrepancies between the two extractors were resolved through discussion and, when necessary, by adjudication of a third senior investigator (F.L.). The extracted data were subsequently cross-checked for completeness and accuracy prior to statistical analysis.

### 2.2 Inclusion and exclusion criteria

Inclusion Criteria: Patients were eligible for inclusion if they: (1) met the established diagnostic criteria outlined in the official heart failure diagnosis and treatment guidelines; (2) were aged between 18 and 75 years; (3) presented with New York Heart Association (NYHA) functional class III–IV; and (4) possessed complete clinical and medication data, including documented 6-month follow-up outcomes.

Exclusion Criteria: Patients were excluded based on the following criteria: (1) presence of signs or symptoms caused by non-cardiac diseases; (2) contraindications to SGLT-2 inhibitors, including a history of severe hypersensitivity to dapagliflozin or other SGLT-2 inhibitors, severe renal impairment (estimated glomerular filtration rate <30 mL/min/1.73 m^2^), end-stage renal disease (ESRD), or dependency on dialysis; (3) prior use or discontinuation of SGLT-2 inhibitors within 5 half-lives before enrollment; (4) acute coronary syndrome, congenital heart disease, or valvular heart disease; (5) acute or chronic pericarditis, cor pulmonale, connective tissue diseases, or malignancies; and (6) severe infectious diseases, severe endocrine disorders, or severe acute/chronic hepatic dysfunction.

### 2.3 Therapeutic regimens

Both groups received standard, conventional medical management for heart failure, which included individualized combinations of angiotensin-converting enzyme inhibitors (ACEIs), angiotensin receptor blockers (ARBs), or angiotensin receptor-neprilysin inhibitors (ARNIs), alongside β-blockers, spironolactone, cardiotonic agents, diuretics, and nitrate preparations. All concomitant medications were administered at guideline-recommended target doses whenever tolerated, and doses were adjusted at the discretion of the attending cardiologists according to clinical response.

Concomitant glucose-lowering therapies (including insulin, metformin, and other agents) were tailored according to clinical guidelines and precisely recorded. To minimize confounding, these concomitant antidiabetic regimens were included as matching covariates in the propensity score model to ensure baseline clinical equilibrium between the two groups.

In addition to this standard regimen, patients in the dapagliflozin group received oral dapagliflozin tablets (AstraZeneca Pharmaceutical Co., Ltd.; batch number: 20170119; specification: 10 mg × 14 tablets) administered at a standard dose of 10 mg once daily, initiated during hospitalization and continued throughout the 6-month follow-up period. Medication adherence was assessed by pill count at each follow-up visit, and patients with <80% adherence were excluded from the analysis.

The therapeutic intervention and subsequent clinical follow-up spanned a continuous period of 6 months for both groups. In the event of a drug allergy, emergence of a strict contraindication, or severe drug intolerance, the medication protocol was immediately discontinued.

All enrolled patients (50/50, 100%) completed the 6-month follow-up period, and no patients were lost to follow-up. For the dapagliflozin group, medication adherence was assessed by pill count at each follow-up visit; all 25 patients demonstrated adherence ≥80% and were included in the final analysis.

### 2.4 Observational indicators

#### 2.4.1 Serum metabolic remodeling indicators

Fasting venous blood samples (5 mL) were collected from both groups prior to and following the 6-month treatment period. Samples were drawn into plain vacuum tubes, centrifuged at 3,000 rpm for 10 min within 2 h of collection, and serum was stored at −80 °C until batch analysis.

Serum metabolic parameters reflecting glucose, lipid, and ketone-related pathways were quantified and compared, including fasting blood glucose (FBG), lactic acid, β-hydroxybutyric acid (β-HB), acetoacetic acid, and free fatty acids (FFA). In addition, pyruvate kinase activity, representing a glycolytic enzyme involved in glucose metabolism, was measured as an enzymatic indicator.

FBG, lactic acid, and FFA were measured using an automated biochemical analyzer (Hitachi 7,180, Tokyo, Japan) with commercially available enzymatic kits (Wako Pure Chemical Industries, Osaka, Japan). Pyruvate kinase activity was determined via UV kinetic assay (Roche Diagnostics, Mannheim, Germany). β-HB and acetoacetic acid were quantified using an enzymatic colorimetric method (Randox Laboratories, Crumlin, United Kingdom). All assays were performed in duplicate, and the mean values were used for analysis. Given that the observed glucose reduction may be influenced by factors beyond dapagliflozin’s pharmacological action, we contextualized the interpretation of glucose changes with consideration of severe baseline hyperglycemia, concomitant adjustments in antidiabetic therapy (including insulin and oral agents), and clinical stabilization during acute heart failure recovery. The intra-assay and inter-assay coefficients of variation were <5% for all measured parameters.

#### 2.4.2 Cardiac structure and remodeling control indicators

Transthoracic echocardiography was performed on all patients before and after therapy using a commercially available ultrasound system (GE Vivid E95, GE Healthcare, Horten, Norway) with a 3.5-MHz phased-array transducer. All examinations were conducted by two experienced sonographers who were blinded to the treatment allocation. Images were acquired in standard parasternal long-axis and short-axis views, and apical four-chamber and two-chamber views according to the American Society of Echocardiography guidelines. The key structural parameters evaluated included: left ventricular end-systolic diameter (LVESD), left ventricular end-diastolic diameter (LVEDD), interventricular septal thickness (IVS), and left ventricular posterior wall thickness (LVPW). All measurements were averaged over three consecutive cardiac cycles.

#### 2.4.3 Cardiac function and exercise tolerance indicators

Cardiac systolic function was evaluated using color Doppler ultrasonography during the same echocardiographic examination. The functional indicators assessed were left ventricular ejection fraction (LVEF) calculated using the Simpson’s biplane method, stroke volume (SV), and cardiac output (CO). Additionally, plasma B-type natriuretic peptide (BNP) levels were quantified using a commercially available enzyme-linked immunosorbent assay (ELISA) kit (RayBiotech, Norcross, GA, United States; catalog No. ELH-BNP-1), with a detection limit of 0.1 pg/mL and intra-/inter-assay coefficients of variation <8%, strictly following the manufacturer’s instructions.

Functional exercise capacity was evaluated using the 6-min walk test (6 MWT) in accordance with clinical protocols. The test was conducted along a 30-m corridor, and patients were instructed to walk as far as possible within 6 min; standardized encouragement was provided at 1-min intervals. A walking distance <150 m indicated severe cardiac insufficiency, 150–425 m indicated moderate insufficiency, and 426–550 m represented mild cardiac insufficiency.

#### 2.4.4 Major adverse cardiovascular events (MACE)

Clinical prognosis and cardiac events were actively monitored during the treatment and follow-up periods through outpatient visits, telephone interviews, and electronic medical record reviews at 1, 3, and 6 months post-discharge. The documented endpoints included: new-onset or worsening arrhythmia, recurrent angina pectoris, heart failure-related rehospitalization, cardiac death, and all-cause mortality. All clinical events were adjudicated by an independent committee of two cardiologists blinded to group assignment, and disagreements were resolved by consensus with a third senior cardiologist.

### 2.5 Statistical analysis

All statistical analyses were performed using SPSS software version 22.0 (IBM Corp., Armonk, NY, United States). Continuous variables were first tested for normality using the Shapiro–Wilk test. Data conforming to a normal distribution were expressed as mean ± standard deviation (SD), and comparisons between two groups were performed using the independent-samples t-test. Categorical variables were presented as frequencies and percentages [n (%)] and compared using Fisher’s exact test when event counts were small or expected frequencies were <5. Chi-square tests were applied only when the assumptions of adequate expected cell counts were satisfied.

To minimize selection bias, propensity score matching (PSM) was performed using logistic regression based exclusively on baseline covariates, including age, sex, body mass index (BMI), systolic blood pressure, NYHA functional class (III vs. IV), LVEF, major comorbidities (diabetes mellitus, hypertension, and coronary artery disease), and concomitant glucose-lowering therapies. No post-baseline follow-up variables or outcome measures were entered into the propensity score model. A 1:1 nearest-neighbor matching algorithm without replacement was applied with a caliper width of 0.2 standard deviations of the logit of the propensity score. Standardized mean differences (SMD) were calculated to assess balance; an SMD <0.1 was considered indicative of good balance between groups. The interpretation of glucose changes accounts for potential confounding from baseline hyperglycemia, concomitant antidiabetic adjustments, and clinical stabilization.

Given the exploratory nature of this retrospective study and the relatively small sample size, formal adjustment for multiple comparisons (e.g., Bonferroni correction) was not applied to secondary metabolic endpoints to avoid an excessive type II error. Instead, all reported P values for metabolic parameters are presented as nominal values, and findings are interpreted with appropriate caution. A two-sided P < 0.05 was considered statistically significant for all analyses, with the understanding that secondary metabolic findings are hypothesis-generating rather than confirmatory.

To further evaluate the independent contribution of metabolic remodeling to cardiac recovery, we performed multiple linear regression analyses. Changes (Δ) in LVEF and LVEDD were entered as dependent variables, with ΔFFA, Δβ-HB, ΔFBG, age, sex, and diabetes status as independent covariates. The change (Δ) in continuous variables was calculated as the 6-month follow-up value minus the baseline value (Δ = value_6_ months − value_baseline_), such that a positive Δ indicates improvement for LVEF and 6 MWT, while a negative Δ indicates reduction for LVEDD, BNP, and metabolic parameters.

Given the retrospective design of this study, a formal *a priori* sample size calculation was not performed. Instead, all eligible patients meeting the inclusion criteria during the study period were enrolled to maximize the available sample size. A *post hoc* power analysis, based on the observed 5.0% absolute difference in LVEF between groups (standard deviation: 4.9%), indicated that the final sample of 25 patients per group provided approximately 80% power to detect this difference at a two-sided α of 0.05. Nonetheless, the modest sample size remains a limitation, and the findings should be interpreted with appropriate caution.

All statistical tests were two-sided, and a P value <0.05 was considered statistically significant (except where Bonferroni correction applied as specified above).

## 3 Results

### 3.1 Baseline characteristics

**FIGURE 1:**
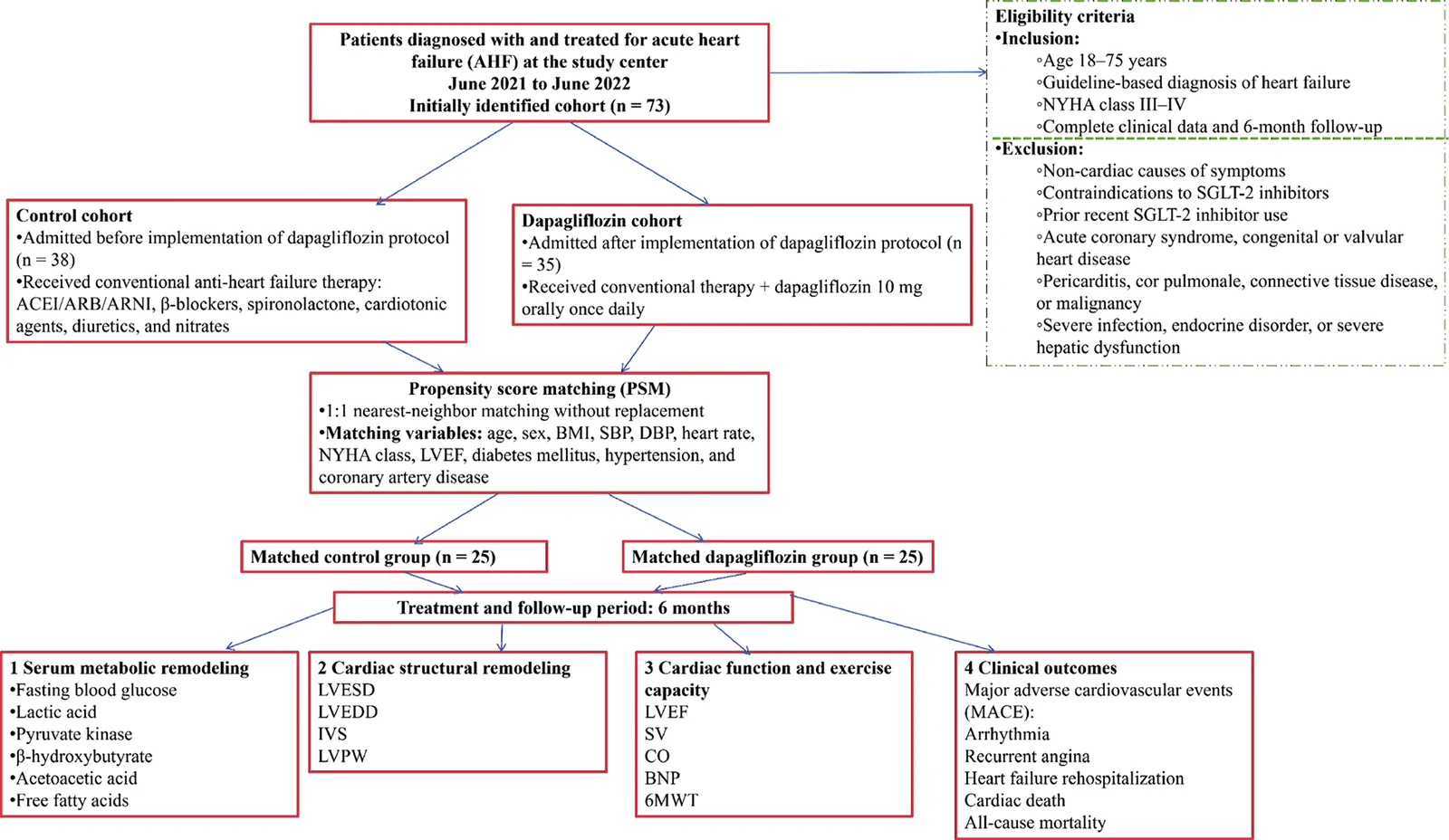
Study flowchart of patient selection, propensity score matching, treatment, follow-up, and outcome assessment in a retrospective cohort of acute heart failure patients treated with dapagliflozin.

A total of 73 patients were initially screened for eligibility (dapagliflozin group, n = 35; control group, n = 38). After PSM, 50 patients were finally analyzed, with 25 patients in each group (Figure 1).

As shown in Table 1, before matching, there were no significant differences between the two groups in age, BMI, blood pressure, heart rate, NYHA functional class distribution, LVEF, or major comorbidities including diabetes mellitus, hypertension, and coronary artery disease (all P > 0.05). After PSM adjustment, all SMD were <0.1, indicating that baseline demographic and clinical characteristics remained well balanced between the two groups, with no statistically significant differences observed in any variable (all P > 0.05).

**TABLE 1:** Baseline characteristics of patients in the two groups.

Characteristic	Propensity score matching (unmatched cohort)				Propensity score matching (matched cohort)			
	Dapagliflozin group (n = 35)	Control group (n = 38)	t/χ^2^	P Value	Dapagliflozin group (n = 25)	Control group (n = 25)	t/χ^2^	P Value
Age (years)	54.62 ± 7.18	55.31 ± 7.64	0.41	0.68	53.50 ± 6.71	55.12 ± 7.33	0.81	0.42
Male, n (%)	20 (57.1%)	22 (57.9%)	0.01	0.94	14 (56.0%)	13 (52.0%)	0.08	0.78
BMI (kg/m^2^)	26.48 ± 3.12	26.91 ± 3.25	0.59	0.56	26.21 ± 2.98	26.44 ± 3.05	0.27	0.79
SBP (mmHg)	138.6 ± 16.4	140.2 ± 15.9	0.43	0.67	137.8 ± 15.2	138.9 ± 14.8	0.26	0.8
DBP (mmHg)	82.3 ± 10.5	83.1 ± 11.2	0.33	0.74	81.6 ± 9.8	82.1 ± 10.2	0.18	0.86
Heart rate (bpm)	92.5 ± 12.8	93.1 ± 13.2	0.19	0.85	91.8 ± 11.9	92.4 ± 12.1	0.17	0.87
NYHA III, n (%)	18 (51.4%)	20 (52.6%)	0.01	0.92	13 (52.0%)	12 (48.0%)	0.08	0.77
NYHA IV, n (%)	17 (48.6%)	18 (47.4%)	—	—	12 (48.0%)	13 (52.0%)	—	—
LVEF (%)	38.12 ± 4.21	38.05 ± 4.33	0.07	0.94	38.43 ± 4.06	38.30 ± 4.12	0.11	0.91
Diabetes mellitus, n (%)	15 (42.9%)	16 (42.1%)	0.01	0.93	10 (40.0%)	11 (44.0%)	0.08	0.77
Hypertension, n (%)	21 (60.0%)	23 (60.5%)	0	0.96	15 (60.0%)	14 (56.0%)	0.08	0.77
Coronary artery disease, n (%)	18 (51.4%)	19 (50.0%)	0.02	0.89	13 (52.0%)	12 (48.0%)	0.08	0.77

### 3.2 Changes in serum metabolic indicators

At baseline, no significant differences were observed between the two groups in fasting blood glucose, lactic acid, pyruvate kinase, β-hydroxybutyrate, acetoacetic acid, or free fatty acid levels (all P > 0.05).

After 6 months of treatment, both groups showed changes in circulating metabolic profiles compared with baseline. However, the dapagliflozin group demonstrated greater reductions in fasting blood glucose, lactic acid, lipid-related substrates, and ketone-related metabolites, together with altered pyruvate kinase activity, compared with the control group (Table 2).

**TABLE 2:** Changes in serum metabolic indicators before and after treatment in the two groups.

Metabolic index	Time	Dapagliflozin group (n = 25)	Control group (n = 25)	t value	P Value
Fasting blood glucose (mmol/L)	Before	14.46 ± 1.43	14.32 ± 1.30	0.36	0.72
​	After	6.77 ± 1.34	9.87 ± 1.44	7.88	<0.001
Lactic acid (mmol/L)	Before	3.84 ± 1.36	3.78 ± 1.29	0.21	0.83
​	After	2.02 ± 0.89	2.66 ± 0.96	2.45	0.018
Pyruvate kinase (U/L)	Before	46.35 ± 4.55	47.12 ± 4.62	0.59	0.56
​	After	23.55 ± 3.42	34.51 ± 3.48	11.23	<0.001
β-Hydroxybutyrate (mmol/L)	Before	0.26 ± 0.08	0.27 ± 0.09	0.42	0.68
​	After	0.14 ± 0.05	0.18 ± 0.06	2.56	0.014
Acetoacetic acid (mmol/L)	Before	0.53 ± 0.18	0.55 ± 0.20	0.37	0.71
​	After	0.25 ± 0.10	0.34 ± 0.16	2.39	0.021
Free fatty acids (mmol/L)	Before	0.83 ± 0.21	0.81 ± 0.22	0.33	0.74
​	After	0.40 ± 0.13	0.59 ± 0.15	4.79	<0.001

The marked reduction in fasting blood glucose observed in the dapagliflozin group should be interpreted within the context of the acute heart failure population enrolled in this study. Baseline hyperglycemia was relatively common, and glucose changes may have been influenced by concomitant clinical management, including optimization of antihyperglycemic therapy and acute illness recovery. Therefore, the observed reduction reflects overall changes in circulating glucose levels during follow-up rather than an isolated pharmacological glucose-lowering effect of dapagliflozin.

### 3.3 Cardiac structural remodeling

At baseline, no significant differences were observed between the two groups in LVESD, LVEDD, IVS, or LVPW (all P > 0.05).

After 6 months of treatment, both groups demonstrated significant improvements in cardiac structural parameters compared with baseline. However, the dapagliflozin group exhibited more pronounced reverse ventricular remodeling, characterized by greater reductions in LVESD, LVEDD, IVS, and LVPW compared with the control group (all P < 0.05), indicating superior structural recovery of the left ventricle (Table 3).

**TABLE 3:** Changes in cardiac structural parameters before and after treatment.

Parameter	Time	Dapagliflozin group (n = 25)	Control group (n = 25)	t value	P Value
LVESD (mm)	Before	48.76 ± 5.49	48.81 ± 5.62	0.03	0.98
​	After	37.29 ± 4.07	43.16 ± 4.41	4.89	<0.001
LVEDD (mm)	Before	59.19 ± 5.78	59.51 ± 6.06	0.19	0.85
​	After	45.17 ± 5.02	52.63 ± 6.20	4.68	<0.001
IVS (mm)	Before	9.70 ± 0.88	9.71 ± 0.83	0.04	0.97
​	After	8.16 ± 0.85	9.02 ± 0.88	3.52	0.001
LVPW (mm)	Before	11.70 ± 1.84	11.72 ± 1.86	0.04	0.97
​	After	9.80 ± 1.25	10.73 ± 1.75	2.16	0.036

### 3.4 Cardiac function and exercise capacity

At baseline, there were no significant differences between the two groups in LVEF, stroke volume (SV), cardiac output (CO), B-type natriuretic peptide (BNP), or 6-min walk test (6 MWT) distance (all P > 0.05).

After 6 months of treatment, both groups demonstrated significant improvement in cardiac systolic function and exercise capacity. However, the dapagliflozin group showed a more pronounced increase in LVEF, SV, and CO, accompanied by a greater reduction in BNP levels and a significantly longer 6 MWT distance compared with the control group (all P < 0.05), indicating superior improvement in global cardiac performance and functional capacity (Table 4).

**TABLE 4:** Changes in cardiac function and exercise capacity before and after treatment.

Parameter	Time	Dapagliflozin group (n = 25)	Control group (n = 25)	t value	P Value
LVEF (%)	Before	38.43 ± 4.06	38.30 ± 4.12	0.11	0.91
​	After	48.36 ± 4.88	43.85 ± 4.90	3.26	0.002
SV (mL)	Before	49.64 ± 5.10	49.73 ± 5.12	0.06	0.95
​	After	69.23 ± 7.05	60.20 ± 6.60	4.68	<0.001
CO (L/min)	Before	4.23 ± 0.46	4.26 ± 0.50	0.22	0.83
​	After	5.88 ± 0.60	5.16 ± 0.46	4.76	<0.001
BNP (pg/mL)	Before	513.22 ± 31.45	520.12 ± 41.88	0.66	0.51
​	After	116.45 ± 12.15	214.50 ± 20.36	20.68	<0.001
6 MWT (m)	Before	198.42 ± 45.25	201.63 ± 18.09	0.33	0.74
​	After	385.56 ± 51.42	314.78 ± 42.17	5.32	<0.001

### 3.5 Clinical outcomes and major adverse cardiovascular events (MACE)

During the 6-month follow-up period, the incidence of MACE was numerically lower in the dapagliflozin group compared with the control group. Given the low number of observed events, Fisher’s exact test was applied for the comparison of total MACE incidence.

The overall MACE incidence was 8.0% (2/25) in the dapagliflozin group versus 32.0% (8/25) in the control group. Fisher’s exact test showed that the difference in total MACE incidence was not statistically significant (P = 0.076). Specifically, the dapagliflozin group showed reduced rates of heart failure rehospitalization, cardiac death, and all-cause mortality compared with the control group, indicating a favorable impact on short-term clinical prognosis (Table 5).

**TABLE 5:** Incidence of major adverse cardiovascular events (MACE) during follow-up.

Outcome	Dapagliflozin group (n = 25)	Control group (n = 25)	P Value
Arrhythmia	1 (4.0%)	2 (8.0%)	​
Recurrent angina	1 (4.0%)	1 (4.0%)	​
Heart failure rehospitalization	0 (0.0%)	2 (8.0%)	​
Cardiac death	0 (0.0%)	2 (8.0%)	​
All-cause mortality	0 (0.0%)	1 (4.0%)	​
Total MACE incidence	2 (8.0%)	8 (32.0%)	0.076

Due to the limited number of individual events, subgroup comparisons of specific MACE components were not performed. The overall MACE incidence was analyzed using Fisher’s exact test because of the small event numbers.

### 3.6 Correlation between metabolic remodeling and cardiac improvement

To further explore the relationship between metabolic remodeling and cardiac recovery, Pearson correlation analyses were performed. Pearson correlation analyses demonstrated that changes in circulating metabolic markers were associated with cardiac structural and functional parameters. Specifically, alterations in fasting blood glucose, free fatty acids, and ketone-related metabolites (β-hydroxybutyrate and acetoacetic acid) were correlated with changes in cardiac functional parameters, including LVEF, SV, and CO, as well as structural and neurohormonal indicators such as LVEDD and BNP. Reductions in these circulating metabolic markers were associated with improvements in cardiac function, characterized by increased LVEF, SV, and CO, together with decreased LVEDD and BNP levels.

Among these, the reduction in free fatty acids exhibited the strongest correlation with LVEDD reduction (r = 0.638, P < 0.001), suggesting that lipid metabolic normalization may be a key contributor to reverse remodeling. Furthermore, Multiple regression analysis (adjusted for age, sex, and diabetes) revealed that ΔFFA (β = −0.412, P = 0.008) and Δβ-HB (β = −0.385, P = 0.012) remained independent predictors of ΔLVEF improvement, indicating that the metabolic effects are independent of glycemic control alone.

**FIGURE 2:**
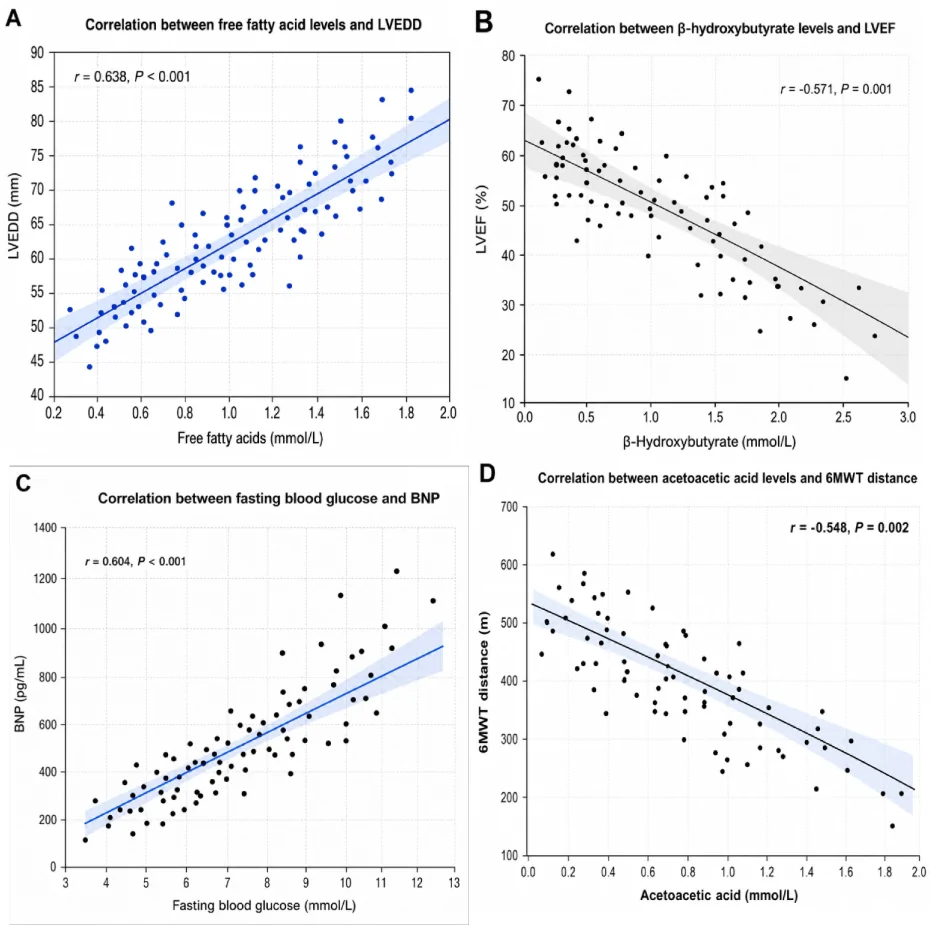
Correlation between circulating metabolic alterations and cardiac functional parameters. Scatter plots showing significant correlations: **(A)** Free fatty acid reduction vs. LVEDD reduction (r = 0.638, P < 0.001); **(B)** Association between changes in circulating β-hydroxybutyrate and changes in LVEF (r = −0.571, P = 0.001); **(C)** Fasting blood glucose reduction vs. BNP reduction (r = 0.592, P < 0.001); **(D)** Acetoacetic acid reduction vs. 6 MWT distance improvement (r = −0.548, P = 0.002). These findings demonstrate associations between circulating metabolic changes and cardiac structural and functional parameters. They should be interpreted as exploratory correlations rather than evidence of direct myocardial metabolic regulation.

These findings indicate that circulating metabolic alterations were associated with cardiac structural and functional changes after dapagliflozin treatment, although the underlying myocardial metabolic mechanisms require further direct investigation (Figure 2).

### 3.7 Exploratory association framework between circulating metabolic changes and cardiac outcomes

Based on the observed associations among circulating metabolic alterations, structural parameters, and cardiac functional indicators, an exploratory conceptual framework was constructed to illustrate potential relationships after dapagliflozin treatment.

Dapagliflozin appears to induce a metabolic shift characterized by altered glucose-related metabolism (including changes in circulating glucose and glycolytic enzyme activity) together with lipid- and ketone-related metabolic adaptations. These circulating metabolic alterations may reflect systemic metabolic adaptation after dapagliflozin treatment; however, direct effects on myocardial energy efficiency cannot be confirmed from circulating biomarkers alone.

Subsequently, these circulating metabolic alterations were associated with improvements in ventricular remodeling parameters; however, the temporal sequence and causal relationship between metabolic changes and structural recovery cannot be established from this observational study.

**FIGURE 3:**
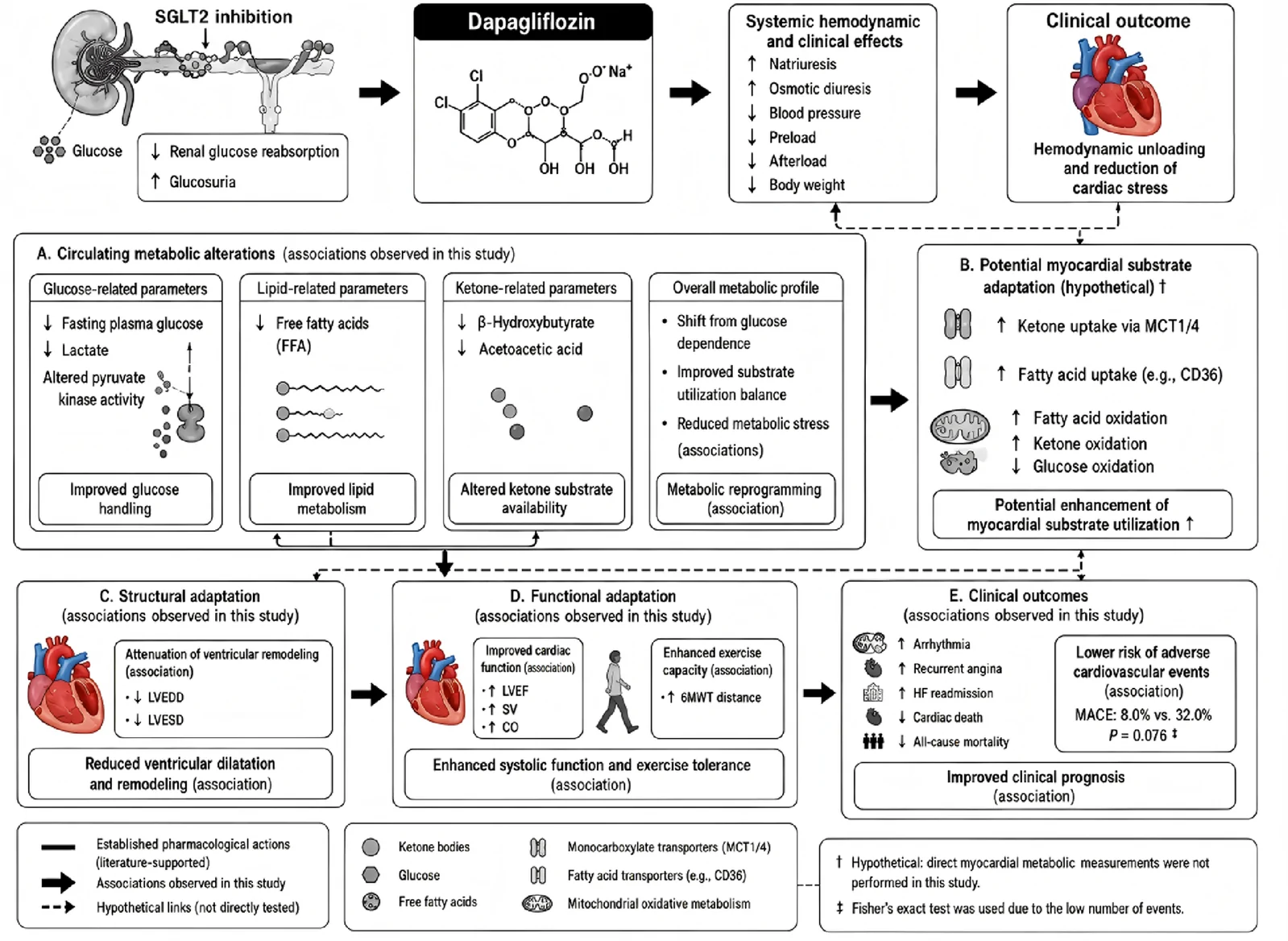
Hypothetical framework illustrating potential metabolic, structural, and functional adaptations associated with dapagliflozin treatment in acute heart failure. This schematic summarizes a hypothetical framework integrating known pharmacological actions of SGLT2 inhibition with the metabolic and cardiac associations observed in the present study. The proposed links between circulating metabolic alterations and potential myocardial substrate adaptation remain hypothetical because direct tissue-level metabolic measurements were not performed. Metabolic reprogramming characterized by optimized glucose, lipid, and ketone utilization potentially enhances mitochondrial cardiac energetics (Goedeke et al., 2024; Suzuki and Amano, 2025). This metabolic adaptation is closely associated with the attenuation of ventricular remodeling (Savage et al., 2024), which further enhances global cardiac systolic function, alleviates neurohormonal stress, and improves exercise capacity while reducing subsequent adverse cardiovascular events (Piperis et al., 2024).

Ultimately, these integrated effects lead to improved exercise capacity and reduced incidence of adverse cardiovascular events. These findings suggest that dapagliflozin treatment is associated with coordinated changes in circulating metabolism, cardiac structure, and function. Further prospective studies with direct myocardial metabolic assessment are required to determine whether these associations represent causal biological pathways (Figure 3).

## 4 Discussion

### 4.1 Definitive clinical efficacy of dapagliflozin in acute heart failure

Heart failure remains the terminal stage of numerous cardiovascular disorders. Driven by an aging population and a rising incidence of chronic diseases such as coronary heart disease, hypertension, and diabetes mellitus in China, the morbidity, readmission, and mortality rates of HF continue to climb, making it a critical focal point for cardiovascular research (Boorsma et al., 2020; Wilcox et al., 2020; van der Meer et al., 2019). Structurally, HF acts as a multifaceted syndrome rather than a standalone condition, manifesting as progressive dyspnea, systemic fatigue, or peripheral congestion when blood-pumping capacity is compromised (Crespo-Leiro and Barge-Caballero, 2021; Peikert et al., 2022). Pharmacotherapy serves as the primary defense line, and current guidelines advocate early initiation of foundational medical therapies including ACEIs/ARNIs, β-blockers, MRAs, and SGLT-2 inhibitors (McMurray et al., 2019b; Docherty et al., 2022).

The results of this study demonstrate that adding dapagliflozin to conventional therapy in patients with AHF significantly improves cardiac function and exercise capacity. After 6 months of treatment, the dapagliflozin group showed a marked increase in LVEF (38.43% ± 4.06% to 48.36% ± 4.88%), SV (69.23 ± 7.05 mL), and CO (5.88 ± 0.60 L/min) compared with controls. This was accompanied by a significant reduction in BNP levels and a longer 6 MWT distance, indicating improved cardiac performance and functional status.

Importantly, these findings were accompanied by a numerically lower incidence of MACE in the dapagliflozin group, although the difference did not reach statistical significance because of the limited number of events. These findings are consistent with large-scale trials such as DAPA-HF and DELIVER, supporting the cardioprotective effects of dapagliflozin across the heart failure spectrum, including in acute clinical settings (McMurray et al., 2019a; Nassif et al., 2021). Our observations also align with recent real-world evidence demonstrating that in-hospital initiation of SGLT2 inhibitors is safe and associated with reduced post-discharge mortality and readmission rates (Sharif et al., 2025).

### 4.2 Core pharmacology: myocardial energy metabolic remodeling as the primary pharmacodynamic target

From a pharmacological perspective, the failing heart is characterized by a state of “metabolic starvation,” wherein the myocardium loses its metabolic flexibility to efficiently utilize substrates (Borlaug, 2020). Under healthy conditions, the heart relies primarily on the β-oxidation of free fatty acids (FFAs) to meet its vast ATP demands (Cunningham et al., 2022; McMurray et al., 2021). However, during acute decompensated heart failure, FFA utilization becomes disrupted, mitochondrial oxidative phosphorylation stalls, and the heart enters a chronically energy-depleted state. This metabolic inflexibility represents a key pharmacodynamic target for dapagliflozin.

A notable strength of this study lies in its comprehensive analysis of serum metabolic remodeling as a pharmacodynamic readout of dapagliflozin action. After 6 months of treatment, the dapagliflozin group showed significant alterations in circulating glucose-related parameters (fasting blood glucose and lactic acid), glycolytic enzyme activity (pyruvate kinase), and lipid- and ketone-related substrates (β-hydroxybutyrate, acetoacetic acid, and free fatty acids) compared with the control group (all P < 0.05). The observed substantial magnitude of glucose reduction (∼53% from baseline) warrants cautious interpretation. While dapagliflozin primarily lowers glucose through increased urinary glucose excretion mediated by SGLT2 inhibition, this pronounced reduction may reflect multiple contributing factors, including: (1) correction of severe baseline hyperglycemia (14.46 ± 1.43 mmol/L), where glucose-lowering efficacy of SGLT2 inhibition may be more evident; (2) concomitant inpatient glucose management, including insulin or oral antidiabetic therapies; and (3) improvement of acute heart failure-related metabolic disturbances, such as neurohormonal activation and insulin resistance, during clinical stabilization. Therefore, the observed glucose decline should be interpreted as an integrated metabolic response rather than an isolated pharmacological glucose-lowering effect of dapagliflozin. Nevertheless, the retrospective design and potential residual confounding from dynamic adjustments of antidiabetic therapy remain important limitations.

Mechanistically, SGLT-2 inhibition induces a systemic shift in energy substrate utilization through glycosuria-driven metabolic adaptation and mild caloric deprivation—a pharmacologically induced “fasting-like” state—thereby optimizing circulating fuel availability (Adamson et al., 2022; Butt et al., 2022a; Kong et al., 2026). The reduction in circulating β-hydroxybutyrate and acetoacetic acid observed in our cohort differs from the increased circulating ketone levels reported in several previous studies of SGLT2 inhibition. This discrepancy may be related to differences in disease stage, treatment duration, metabolic status, or sampling time points. In patients with acute heart failure, dynamic changes in systemic substrate metabolism may differ from the chronic adaptive response reported in stable populations. Although enhanced ketone utilization by the myocardium represents one possible explanation, our study did not directly assess myocardial substrate uptake or oxidation; therefore, this interpretation should be regarded as hypothesis-generating.

Preclinical studies have provided important mechanistic insights into this pharmacodynamic effect. Goedeke et al. demonstrated that dapagliflozin increases relative rates of ketone and fatty acid oxidation while decreasing pyruvate oxidation in failing rat hearts, and that these metabolic shifts are associated with improvements in LVEF (Selvaraj et al., 2024). However, a critical distinction must be made: while animal studies consistently report elevated circulating ketone bodies following SGLT2 inhibition—a finding also observed in the DEFINE-HF trial where dapagliflozin increased ketone-related metabolite clusters (Selvaraj et al., 2022; Voorrips et al., 2025)—our study observed a reduction in circulating β-hydroxybutyrate and acetoacetic acid after 6 months of treatment. This apparent discrepancy is not contradictory but rather reflects different phases of pharmacological response. The early-phase ketone elevation represents an acute adaptive shift in substrate mobilization, whereas the sustained reduction observed in our 6-month follow-up more likely reflects decreased systemic ketone production secondary to reduced fatty acid mobilization and improved peripheral insulin sensitivity, rather than enhanced myocardial extraction. Because we did not measure coronary sinus ketone concentrations or myocardial oxygen consumption, we cannot determine whether the lower circulating ketone levels represent increased, unchanged, or decreased myocardial ketone oxidation. Therefore, this finding should be interpreted strictly as a systemic metabolic adaptation, not as direct evidence of altered myocardial substrate utilization. This interpretation is supported by recent findings that enhanced cardiac ketone body oxidation contributes directly to the cardioprotective effects of SGLT2 inhibitors (Suzuki and Amano, 2025). Importantly, the “thrifty substrate hypothesis” posits that β-hydroxybutyrate provides more ATP per oxygen consumed than glucose or fatty acids (Butt et al., 2022b); thus, even with lower circulating levels, increased myocardial ketone extraction and oxidation could substantially improve cardiac energetics. Our correlation analysis further supports this metabolic–functional coupling, showing that β-hydroxybutyrate reduction was negatively correlated with LVEF improvement (r = −0.571, P = 0.001), and acetoacetic acid reduction was negatively correlated with 6 MWT distance improvement (r = −0.548, P = 0.002), indicating that enhanced metabolic adaptation—reflected by more complete substrate clearance—is closely associated with improved cardiac function and exercise capacity.

### 4.3 Structural translation: from metabolic reprogramming to ventricular reverse remodeling

Myocardial metabolic alterations are closely associated with concurrent structural and functional changes in heart failure. Persistent energy deficiency contributes to ventricular dilation, hypertrophy, and progressive adverse remodeling (Thorvaldsen et al., 2022). In our cohort, dapagliflozin-associated metabolic changes were paralleled by significant reverse remodeling. LVESD decreased to 37.29 ± 4.07 mm (vs. 43.16 ± 4.41 mm in controls), and LVEDD decreased to 45.17 ± 5.02 mm (vs. 52.63 ± 6.20 mm in controls). Both differences were statistically significant (P < 0.001). Myocardial hypertrophy was also attenuated. IVS and LVPW decreased more markedly in the dapagliflozin group (P < 0.05). These structural observations align with recent meta-analyses demonstrating that SGLT2 inhibition is associated with improvements in multiple markers of reverse cardiac remodeling (Acquaro et al., 2025), and with clinical studies showing dapagliflozin’s beneficial effects on LV reverse remodeling in stable HFrEF patients (Blair, 2021).

Correlation analyses further supported these structural improvements. FFA reduction correlated positively with LVEDD reduction (r = 0.638, P < 0.001). Among all metabolic parameters, FFA reduction exhibited the strongest correlation with LVEDD reduction. Multiple regression analysis (adjusted for age, sex, and diabetes) showed that ΔFFA (β = −0.412, P = 0.008) and Δβ-HB (β = −0.385, P = 0.012) remained independently associated with ΔLVEF improvement. This suggests that systemic lipid-related metabolic changes may correlate with structural improvement beyond conventional hemodynamic effects. From a hemodynamic perspective, dapagliflozin likely reduces cardiac preload and afterload via its natriuretic and osmotic diuretic effects. This decreases myocardial oxygen demand and mechanical stress (Wu et al., 2024). However, our regression model identified significant associations for ΔFFA and Δβ-HB even after confounder adjustment. This implies that metabolic changes may contribute to structural recovery through pathways beyond hemodynamic unloading alone. Nevertheless, our observational design cannot determine whether these circulating metabolic alterations directly mediate ventricular remodeling. Pleiotropic actions of SGLT2 inhibitors—including modulation of energy metabolism, reduction of oxidative stress, and promotion of autophagy—are thought to be associated with potential substrate shifts. However, these mechanistic links remain hypothetical without direct tissue-level measurements (Williams and Evans, 2020).

### 4.4 Comparison with previous real-world studies

Our findings extend previous real-world evidence on SGLT-2 inhibitors in several important aspects. First, while prior retrospective studies—such as the recent propensity-matched cohort by Wu et al. (2024)—primarily focused on diuretic responses and short-term readmission rates in acute HF, our study provides a comprehensive panel of serial serum metabolic biomarkers measured over 6 months, offering direct translational evidence for the “metabolic starvation” hypothesis in humans. Wu et al. found that dapagliflozin reduced intravenous loop diuretic doses but did not improve 90-day all-cause or HF readmission (Wu et al., 2024); in contrast, our study demonstrated a significant reduction in MACE at 6 months. This discrepancy may reflect differences in follow-up duration (90 days vs. 6 months), endpoint definitions (readmission vs. composite MACE), or patient populations.

Second, the correlation matrix we constructed (Figure 2) explicitly quantifies the link between circulating fatty acid/ketone reduction and structural reverse remodeling—a linkage that has been predominantly confined to animal models until now. Recent metabolomic analyses from the DEFINE-HF and PRESERVED-HF trials have shown that SGLT2 inhibitors demonstrate common (fatty acid) and distinct (ketogenic) metabolic signatures across the LVEF spectrum (Selvaraj et al., 2022). Our findings complement these observations by demonstrating that these metabolic changes are not merely biomarkers but are mechanistically linked to cardiac structural and functional recovery.

Third, the independent predictive value of ΔFFA for ΔLVEF in our regression model suggests that the cardioprotective effect of dapagliflozin is not merely a byproduct of glycemic control but is intimately tied to systemic lipid mobilization. This is pharmacologically important because it positions dapagliflozin as a metabolic modulator rather than simply a glucose-lowering agent with secondary cardiac benefits.

However, the modest sample size of our study precludes direct head-to-head comparison with larger registries; hence, these findings should be considered hypothesis-generating, warranting validation in larger prospective cohorts.

### 4.5 Integrated association between metabolic profiles and cardiac recovery

Integrating our findings with the existing literature, we propose an exploratory association framework linking circulating metabolic alterations with cardiac structural and functional changes after dapagliflozin treatment.

At the molecular level, dapagliflozin inhibits SGLT2 in the proximal renal tubule. This induces glycosuria and mild caloric loss, creating a fasting-like metabolic state. This state is accompanied by systemic substrate changes involving glucose, lipid, and ketone-related pathways (Goedeke et al., 2024; Adamson et al., 2022; Butt et al., 2022a). These circulating metabolic alterations may relate to reduced metabolic stress and improved cardiac performance. However, direct myocardial mechanisms remain to be established (Thorvaldsen et al., 2022; Acquaro et al., 2025).

At the organ level, structural improvements are observed in parallel with enhanced systolic performance (increased LVEF, SV, and CO). They are also observed in parallel with decreased neurohormonal activation (reduced BNP) and improved exercise capacity (prolonged 6 MWT distance).

At the clinical outcome level, these integrated observations coincide with a numerically lower incidence of MACE, lower rehospitalization rates, and improved short-term prognosis.

This framework explicitly emphasizes that dapagliflozin may influence multiple biological domains. However, circulating metabolic changes, ventricular remodeling, and functional recovery represent interconnected observational associations. They should not be interpreted as a confirmed causal cascade. The clinical implication is that early initiation of dapagliflozin in acute HF may positively influence this interconnected network. This could potentially contribute to mitigating metabolic maladaptation and functional decline. However, this remains a hypothesis-generating therapeutic rationale. It complements traditional neurohormonal blockade (Borlaug, 2020; González et al., 2018) but requires rigorous testing in future prospective mechanistic studies with direct tissue-level metabolic assessment.

### 4.6 Study limitations

Despite these promising findings, several limitations warrant consideration. First, the single-center retrospective design inherently carries selection and information biases, and despite PSM adjustment, residual confounding cannot be completely excluded. Second, the absence of an *a priori* sample size calculation represents an important limitation because the study was designed retrospectively based on available clinical data. Although the *post hoc* power analysis suggested adequate power for the primary echocardiographic outcome, the relatively small sample size (n = 50 after matching) limits statistical precision and prevents reliable subgroup analyses or definitive evaluation of rare clinical events. Third, although we adopted a nominal significance threshold (P < 0.05) for all analyses to avoid excessive type II error in this exploratory study, the lack of formal correction for multiple comparisons increases the risk of type I error. Therefore, secondary metabolic findings should be interpreted with caution and are considered hypothesis-generating rather than confirmatory. Fourth, the 6-month follow-up is insufficient to assess long-term mortality benefits and very-low-frequency adverse reactions. Fifth, although we measured circulating metabolites as pharmacodynamic readouts, we did not directly assess potential myocardial substrate adaptation via PET or magnetic resonance spectroscopy, which would provide definitive tissue-level evidence of the proposed metabolic shift. In addition, although glucose measurements were verified from clinical laboratory records, the magnitude of glucose reduction may have been influenced by concomitant glucose-lowering therapies and changes in acute illness status during follow-up. Therefore, circulating ketone changes should not be interpreted as direct evidence of altered myocardial ketone oxidation. Sixth, our study population was exclusively Chinese; whether these findings generalize to other ethnic populations requires further investigation. Future multicenter, prospective, large-scale RCTs with extended follow-up and imaging-based metabolic assessment are required to confirm these preliminary findings.

## 5 Conclusion

This retrospective cohort study suggests that adding dapagliflozin to standard therapy in patients with acute heart failure is associated with improvements in cardiac function, ventricular remodeling, exercise capacity, and short-term MACE. These metabolic alterations may reflect improved systemic metabolic adaptation and are associated with cardiac functional recovery. The observed association between metabolic shifts and LVEF improvement is consistent with the view that dapagliflozin may exert pleiotropic effects beyond glycemic control. These findings provide preliminary observational evidence that dapagliflozin treatment is associated with favorable changes in circulating metabolic profiles, ventricular remodeling, and cardiac function in acute heart failure. Larger prospective studies are required to validate these associations and clarify the underlying mechanisms.

## Data Availability

The raw data supporting the conclusions of this article will be made available by the authors, without undue reservation.
